# Monkfish Peptides Mitigate High Fat Diet-Induced Hepatic Steatosis in Mice

**DOI:** 10.3390/md20050312

**Published:** 2022-05-05

**Authors:** Jiena Ye, Xiaoxiao Tian, Qiongfen Wang, Jiawen Zheng, Yanzhuo Yang, Baogui Xu, Shuai Zhang, Falei Yuan, Zuisu Yang

**Affiliations:** 1 Zhejiang Provincial Engineering Technology Research Center of Marine Biomedical Products, School of Food and Pharmacy, Zhejiang Ocean University, Zhoushan 316022, China; z20105500018@zjou.edu.cn (J.Y.); z18095135044@zjou.edu.cn (X.T.); jwzheng1996@163.com (J.Z.); z20105500026@zjou.edu.cn (Y.Y.); z19105500022@zjou.edu.cn (B.X.); zhangxj_hys@163.com (S.Z.); 2 Zhoushan Institute for Food and Drug Control, Zhoushan 316000, China; wqf6512@aliyun.com

**Keywords:** monkfish peptides, NAFLD, the AMPK/Nrf2 pathway, lipid metabolism

## Abstract

Non-alcoholic fatty liver disease (NAFLD) is a hepatic metabolic syndrome usually accompanied by fatty degeneration and functional impairment. The aim of the study was to determine whether monkfish peptides (LPs) could ameliorate high-fat diet (HFD)-induced NAFLD and its underlying mechanisms. NAFLD was induced in mice by giving them an HFD for eight weeks, after which LPs were administered in various dosages. In comparison to the HFD control group: body weight in the LP-treated groups decreased by 23–28%; triacylglycerol levels in the blood decreased by 16–35%; and low-density lipoproteins levels in the blood decreased by 23–51%. Additionally, we found that LPs elevated the activity of hepatic antioxidant enzymes and reduced the inflammatory reactions within fatty liver tissue. Investigating the effect on metabolic pathways, we found that in LP-treated mice: the levels of phospho-AMP-activated protein kinase (p-AMPK), and phospho-acetyl CoA carboxylase (p-ACC) in the AMP-activated protein kinase (AMPK) pathway were up-regulated and the levels of downstream sterol regulatory element-binding transcription factor 1 (SREBP-1) were down-regulated; lipid oxidation increased and free fatty acid (FFA) accumulation decreased (revealed by the increased carnitine palmitoyltransferase-1 (CPT-1) and the decreased fatty acid synthase (FASN) expression, respectively); the nuclear factor erythroid-2-related factor 2 (Nrf2) antioxidant pathway was activated; and the levels of heme oxygenase-1 (HO-1) and nicotinamide quinone oxidoreductase 1 (NQO1) were increased. Overall, all these findings demonstrated that LPs can improve the antioxidant capacity of liver to alleviate NAFLD progression mainly through modulating the AMPK and Nrf2 pathways, and thus it could be considered as an effective candidate in the treatment of human NAFLD.

## 1. Introduction

Non-alcoholic fatty liver disease (NAFLD) refers to a clinicopathological syndrome characterized by inflammation of the liver lobule and hepatic parenchymal steatosis. NAFLD can range from simple steatosis to more severe liver diseases, such as steatohepatitis, fibrosis, and even liver cancer [1]. The occurrence of NAFLD worldwide is between 14% and 24% of liver diseases, yet the underlying mechanism of the condition remains unclear [2]. HFD provides a high amount of saturated fatty acids, especially palmitic acid (C16:0). The excess of palmitic acid generates an increase in the hepatic inflammatory response [3]. With a lack of clinical intervention options, diet regulation remains the most effective means of managing NAFLD [4].

Recent research has focused largely on the effects of mitochondrial dysfunction, insulin resistance, obesity, and changes to the gut microbiome on the development of NAFLD. Mitochondrial dysfunction is involved in the progression of NAFLD, which changes the homeostasis of fatty liver, thereby producing higher levels of malondialdehyde (MDA) and reactive oxygen species (ROS) [5,6]. When oxidative stress occurs, nuclear factor erythroid-2-related factor 2 (Nrf2) combines with antioxidant redox elements to protect cells against stress. Together, they regulate the expression of downstream genes such as heme oxygenase-1 (HO-1) and nicotinamide quinone oxidoreductase 1 (NQO1) [7,8]. Both liraglutide and tetrahydrocurcumin ameliorated NAFLD by increasing the mRNA levels of Nrf2, HO-1, GCLM, and NQO1 [9,10]. The body’s antioxidant defense enzymes include catalase (CAT), glutathione peroxidase (GSH-Px), and superoxide dismutase (SOD). Decreasing these enzymes can also lead to oxidative stress.

Insulin resistance breaks down the adipose tissue, which in turn releases more free fatty acids (FFA), adipokines, and inflammatory factors to the liver and adipose tissue. Much attention has been paid to reducing the level of FFA in the treatment of NAFLD [11]. AMP-activated protein kinase (AMPK) is recognized as a major energy-related protein kinase that mediates the development of liver dysfunction [11]. Acetyl CoA carboxylase (ACC) is the target molecule of AMPK. ACC catalyzes the production of malonyl-CoA by prompting the synthesis and elongation of fatty acids as well as inhibiting the oxidation of fatty acids through the inhibition of carnitine palmitoyltransferase-1 (CPT-1) [12]. AMPK phosphorylation can induce ACC phosphorylation and inhibit ACC activity. ACC enters a negative feedback loop that increases the content of CPT-1 and oxidizes fatty acids, thus reducing fatty acid content in the liver [13]. The activation of AMPK weakens the proteolytic process of sterol regulatory element-binding proteins (SREBP)-1c and leads to accelerated fatty acid oxidation, thereby eliminating the abnormal accumulation of FFA. Liensinine significantly ameliorated HFD-triggered hepatic oxidative stress and dyslipidemia by mediating Nrf2/AMPK signaling, and dimethyl fumarate similarly mitigated NAFLD progression by mediating Nrf2, SREBP-1c, and nuclear factor-κB (NF-κB) signaling [14,15]. Excessive dietary fat directly enhances hepatic lipid synthesis and inflammatory responses [16,17]. Obesity is closely related to NAFLD, because it is characterized by liver-neutral lipid accumulation [18,19]. One of the morphological features of steatosis is the accumulation of lipid droplets in hepatic parenchymal cells.

Bioactive peptides, generally composed of three to twenty amino acid residues, are known for their antihypertensive and antilipemic properties [20]. Milk is recognized as a major source of bioactive peptides and fish proteins may be another important source [21]. Although it has been shown that protein hydrolysates from yellow catfish alleviate mouse NAFLD through inhibiting lipid metabolism, the underlying mechanisms have yet to be studied [22]. Herring milt protein hydrolysates attenuate insulin resistance from excessive fat consumption [23]. Fish protein hydrolysates can regulate lipid metabolism and improve dyslipidemia in Zucker rats [24,25]. Every year, approximately 12% of aquatic products worldwide are used for non-food purposes [24]. Monkfish meat has traditionally been considered a low-value food. Monkfish peptides (LPs) have been found to have strong antioxidant activities and fat absorption capabilities [26,27,28]. However, the regulatory effects of LPs on NAFLD, and the underlying molecular mechanisms are still unclear. Thus, in the current study, we tested the protective effects of LPs against high-fat diet-induced NAFLD in mice. We also sought to determine whether the protective effects of LPs involve the AMPK and Nrf2 signaling pathways.

## 2. Results

### 2.1. Identification and Protein Analysis of LPs

LPs with a molecular weight of less than 1 kDa were harvested, and 198 mg of LPs were prepared from 100 g of the fish meat. The total yield was 0.198%. Using database alignment, the corresponding sequences were found in the protein database of the Lophiiformes species in the National Center for Biotechnology Information (NCBI). As shown in Supplementary Table S1, database matching obtained 98 low molecular weight peptides that consisted of 17 heptapeptides, 31 octapeptides, 26 nonapeptides, 13 decapeptides, 9 undecapeptides, and 2 dodecapeptides. There were 96 peptides with two charges and 2 peptides with one charge. A total of 3276 free peptides were identified by de novo sequencing, and the results did not overlap with the data obtained through the database comparison method. The shortest free peptide identified through de novo sequencing was a tetrapeptide and the longest was an octapeptide. Amino acid local confidence (ALC) is the confidence level of de novo sequencing data. Generally, ALC > 80% is adequately reliable. Accordingly, Supplementary Table S2 lists 66 free peptides with ALC > 80% identified by the de novo sequencing results. They had a distribution ranging from tetrapeptide to nonapeptide, including 13 tetrapeptides, 32 pentapeptides, 13 hexapeptides, 4 heptapeptides, and 4 nonapeptides. Additionally, 39 polypeptides had two charges and 27 polypeptides had one charge. Supplementary Table S3 lists the types of proteins. Each of these proteins plays a different role in organisms. For example, the protein nicotinamide adenine dinucleotide (NADH) dehydrogenase is an enzyme located in the inner membrane of mitochondria that catalyzes the transfer of electrons from NADH to coenzyme Q. This enzyme is an “entry enzyme” of oxidative phosphorylation in the mitochondria [29]. ATPase, also known as adenosine triphosphatase, catalyzes the hydrolysis of adenosine triphosphate (ATP) to adenosine diphosphate (ADP) and a phosphate ion. Cytochrome c oxidase transfers the electron of a respiratory substrate directly to molecular oxygen (i.e., with automatic oxidation) through the cytochrome system. This shows that there is an abundance of low-molecular-weight peptides in LPs, of which tetrapeptides and pentapeptides account for most. These protein types also show that LPs are likely to have good antioxidant activity, which is consistent with the results of previous studies [27].

### 2.2. LPs Lowered Body Weight and Liver Weight

**Figure 1:**
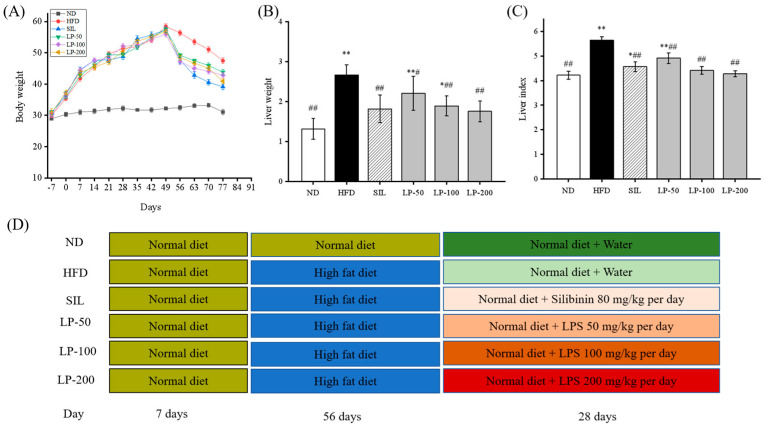
(**A**): Effects of LPs on the alterations of body weight. (**B**): Effects of LPs on the alterations of liver weight. (**C**): Effects of LPs on the liver index (Liver weight/Body weight × 100) of mice with non-alcoholic fatty liver disease (NAFLD). (**D**): Research schedule of NAFLD mice. Values are presented as means ± SD. Values with different labels are significantly different in the groups (* *p* < 0.05, ** *p* < 0.01 vs. ND group, ^#^ *p* < 0.05, ^##^ *p* < 0.01 vs. HFD group).

As shown in Figure 1A, after seven days of adaptive feeding, the mice in the experimental groups were fed with a high-fat diet (HFD) for eight weeks. The average body weight of mice in the HFD group increased 82% more than that of the normal diet (ND) group. From Week 9 onward, all groups were fed normal diets (Figure 1D) during which the average weight of all the experimental groups decreased (LP-50, 24%, LP-100, 23%, LP-200, 28%, and silibinin (SIL) 32%). After another four weeks of intervention, the average liver weight and liver weight index of the HFD group, calculated as the percentage of liver weight divided by body weight, was higher than that of the ND group by 51% and 34% (*p* < 0.01 Figure 1B,C). Meanwhile the average liver weight and liver weight indexes of the SIL, LP-50, LP-100, and LP-200 groups decreased by 32%, 17%, 29%, and 34%, and 19%, 13%, 22%, and 25%, respectively, as compared with the HFD group (*p* < 0.01 Figure 1B,C).

### 2.3. Effects of LPs on Blood Lipids

**Figure 2:**
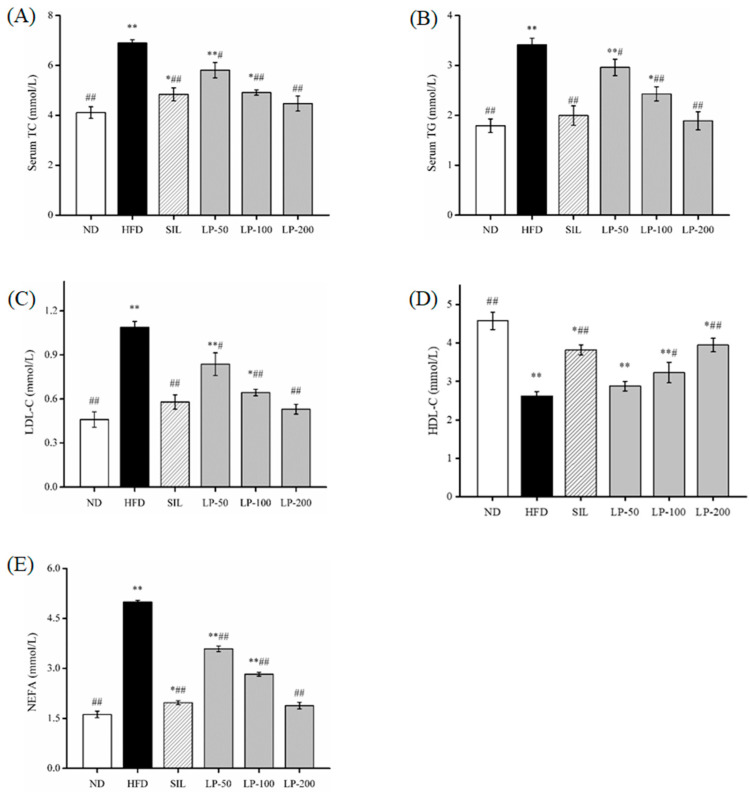
Effects of LPs on plasma total cholesterol (TC) (**A**), triacylglycerol (TG) (**B**), low-density lipoprotein (LDL-c) (**C**), high-density lipoprotein (HDL-c), (**D**) and free fatty acids (FFA) (**E**). Values are presented as means ± SD. Values with different labels are significantly different in the groups (* *p* < 0.05, ** *p* < 0.01 vs. ND group, ^#^ *p* < 0.05, ^##^ *p* < 0.01 vs. HFD group).

The results of the blood lipid indexes found in the serum are shown in Figure 2. Compared to the ND group serum, total cholesterol (TC) levels increased by a substantial 68% in the HFD group (*p* < 0.01, Figure 2A). Meanwhile the SIL and LPs-treated groups (LP-50, LP-100, and LP-200) decreased by 30%, 16%, 29%, and 35%, respectively, in contrast to the HFD group (*p* < 0.05, Figure 2A). There was no significant difference between the LP-200 group and the ND group, and the repair effect was the best between them. The changes in triacylglycerol (TG) and low-density lipoproteins (LDL)-c levels in the serum were the same. In comparison with the ND group, the TG and LDL-c concentrations in the HFD group increased by 91% and 137%, respectively (*p* < 0.01, Figure 2B,C). Compared with the HFD group, the concentration of TG decreased in the SIL, LP-50, LP-100, and LP-200 groups by 42%, 13%, 29%, and 45%, respectively. Furthermore, the concentration of LDL-c decreased in the SIL, LP-50, LP-100, and LP-200 groups by 47%, 23%, 41%, and 51%, respectively (*p* < 0.05, Figure 2B,C). The concentrations of TG and LDL-c in the SIL group and the LP-200 group were not significantly different from the ND group. Compared with the ND group, the concentration of high-density lipoproteins (HDL)-c in each experimental group decreased significantly (*p* < 0.05, Figure 2D). Compared with the HFD group, the level of HDL-c in the LP-50 group was not significantly different, and the level of HDL-c in the SIL, LP-100, and LP-200 groups increased by 46%, 9%, 23%, and 50%, respectively (*p* < 0.05, Figure 2D). The FFA levels in the serum of the HFD group were twice the amount of that in the ND group (*p* < 0.01, Figure 2E). The FFA levels in the serum decreased in the SIL, LP-50, LP-100, and LP-200 by 61%, 28%, 43%, and 62%, respectively, compared to the HFD group (*p* < 0.01, Figure 2E). There was no significant difference between the LP-200 group and the ND group, and the repair effect was the best in the LP-200 group. Thus, it can be concluded that the LP-200 group is most effective in regulating blood lipid levels.

### 2.4. Effects of LPs on Aspartate Aminotransferase (AST) and Alanine Aminotransferase (ALT)

**Figure 3:**
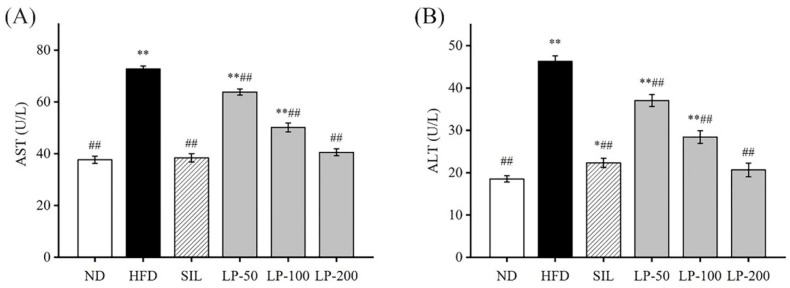
Effects of LPs on the levels of aspartate aminotransferase (AST) (**A**) and alanine aminotransferase (ALT) (**B**). Values with different labels are significantly different in the groups (* *p* < 0.05, ** *p* < 0.01 vs. ND group, ^##^ *p* < 0.01 vs. HFD group).

An increase in aspartate aminotransferase (AST) and alanine aminotransferase (ALT) activity in the blood is usually interpreted as a marker for lesions [30]. The ALT and AST levels in the HFD group increased by 105% and 93%, respectively, indicating that an HFD gave rise to liver injuries (*p* < 0.01, Figure 3). Compared with the HFD group, ALT contents in the serum of the SIL, LP - 50, LP-100, and LP-200 groups decreased by 52%, 20%, 37%, and 55%, respectively, and the concentration of AST in the SIL, LP-50, LP-100, and LP-200 decreased by 47%, 12%, 31%, and 44%, respectively (*p* < 0.01, Figure 3). Although no statistical significance was found among the SIL, LP-200, and ND groups, AST levels in the LP-50 and the LP-100 groups were significantly higher than baseline, a trend similarly followed by ALT levels. The ALT levels of LP-50 and LP-100 were significantly higher than those of the ND group (*p* < 0.01 Figure 3B), and so were the levels in the SIL group (*p* < 0.05 Figure 3B). The LP-200 group did not show any significant difference.

### 2.5. Effects of LPs on Antioxidant Capacity

The antioxidant capacity of liver tissues in different groups is shown in Table 1. The levels of total antioxidant capacity (T-AOC), CAT, SOD, and GSH-Px in the HFD group were significantly lower than those in the ND group (*p* < 0.01). The levels of these same indicators in the SIL and LPs-treated groups were significantly lower than those in the HFD group (*p* < 0.01). There was no significant difference between the SIL, LP-200, and ND groups. The MDA level in the HFD group was significantly higher than that in the ND group. Compared with the HFD group, the MDA content in the SIL, LP-100, and LP-200 groups significantly decreased (*p* < 0.05).

### 2.6. Effects of LPs on the Level of Inflammatory Factors

**Figure 4:**
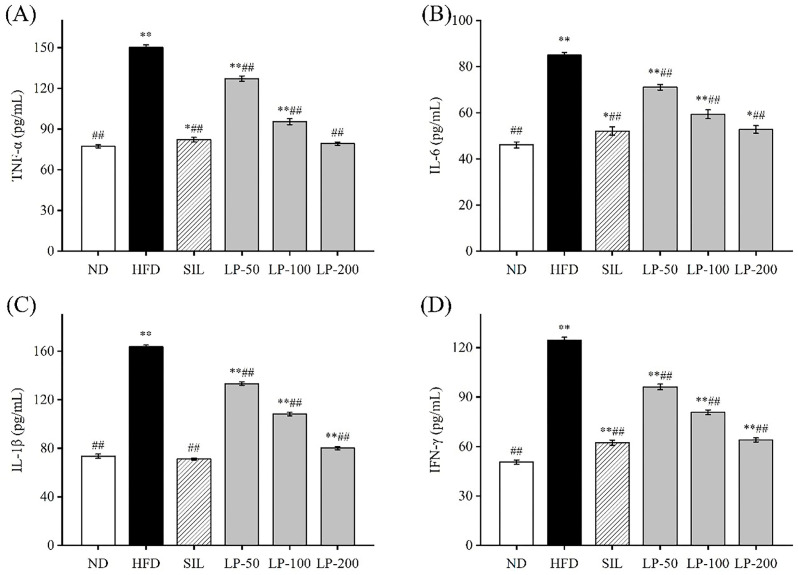
Effects of LPs on the level of inflammatory factors: TNF-α (**A**), IL-6 (**B**), IL-1β (**C**), and IFN-γ (**D**). Data are presented as means ± SD. Values with different labels are significantly different among the groups (* *p* < 0.05, ** *p* < 0.01 vs. ND group, ^##^ *p* < 0.01 vs. HFD group).

To determine the effects of LPs on inflammation, we examined the levels of IL-6, IL-1β, TNF-α, and IFN-γ in the blood. The concentration of TNF-α in the HFD group increased 94% in comparison with the ND group (*p* < 0.01, Figure 4A). The concentration of TNF-α in the SIL, LP-50, LP-100, and LP-200 groups decreased by 42%, 18%, 36%, and 45%, respectively, compared to the HFD group levels (*p* < 0.01, Figure 4A). There was no significant difference between the LP-200 group and the ND group. The concentration of IL-6 in each experimental group significantly increased in comparison to the ND group (*p* < 0.05, Figure 4B). The concentration of IL-6 in the SIL, LP-50, LP-100, and LP-200 groups decreased by 38%, 14%, 26%, and 39%, respectively, compared with the HFD group (*p* < 0.01, Figure 4B). The concentrations of IL-1β and IFN-γ in the HFD group were significantly higher than those in the ND group (*p* < 0.01, Figure 4C,D). Compared with the HFD group, IL-1β levels in the SIL, LP-50, LP-100, and LP-200 groups showed significant decreases of 46%, 16%, 40%, and 45%, respectively, and as did the levels of IFN-γ in these groups with decreases of 45%, 17%, 42%, and 51%, respectively (*p* < 0.01, Figure 4C,D). From these results, it can be concluded that the SIL group and the LP-200 group were the best at inhibiting the inflammatory response of mice with NAFLD.

### 2.7. Effects of LPs on Hepatic Histopathology

**Figure 5:**
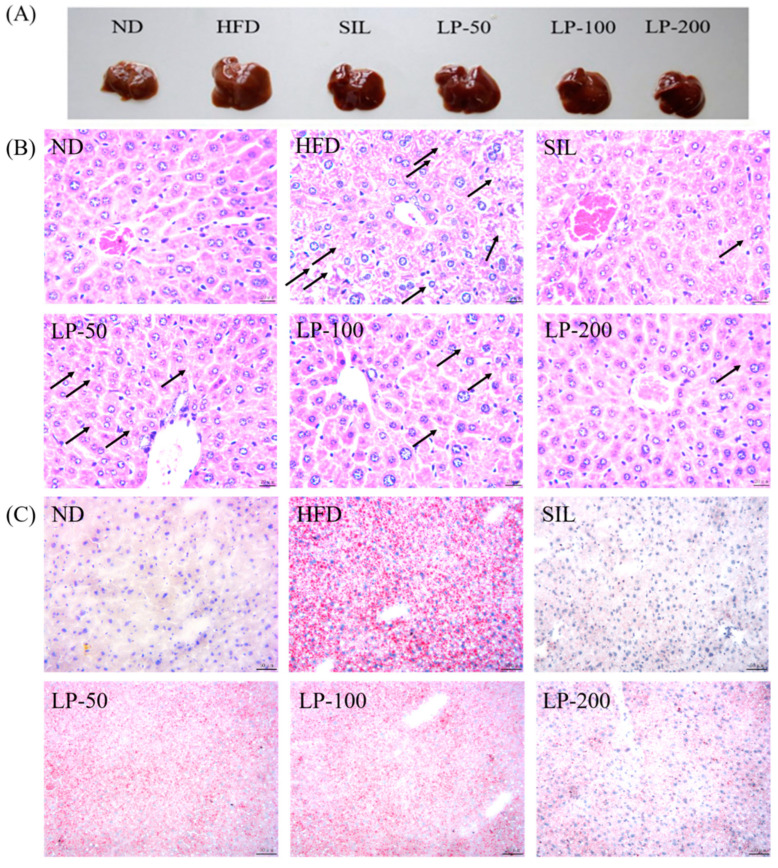

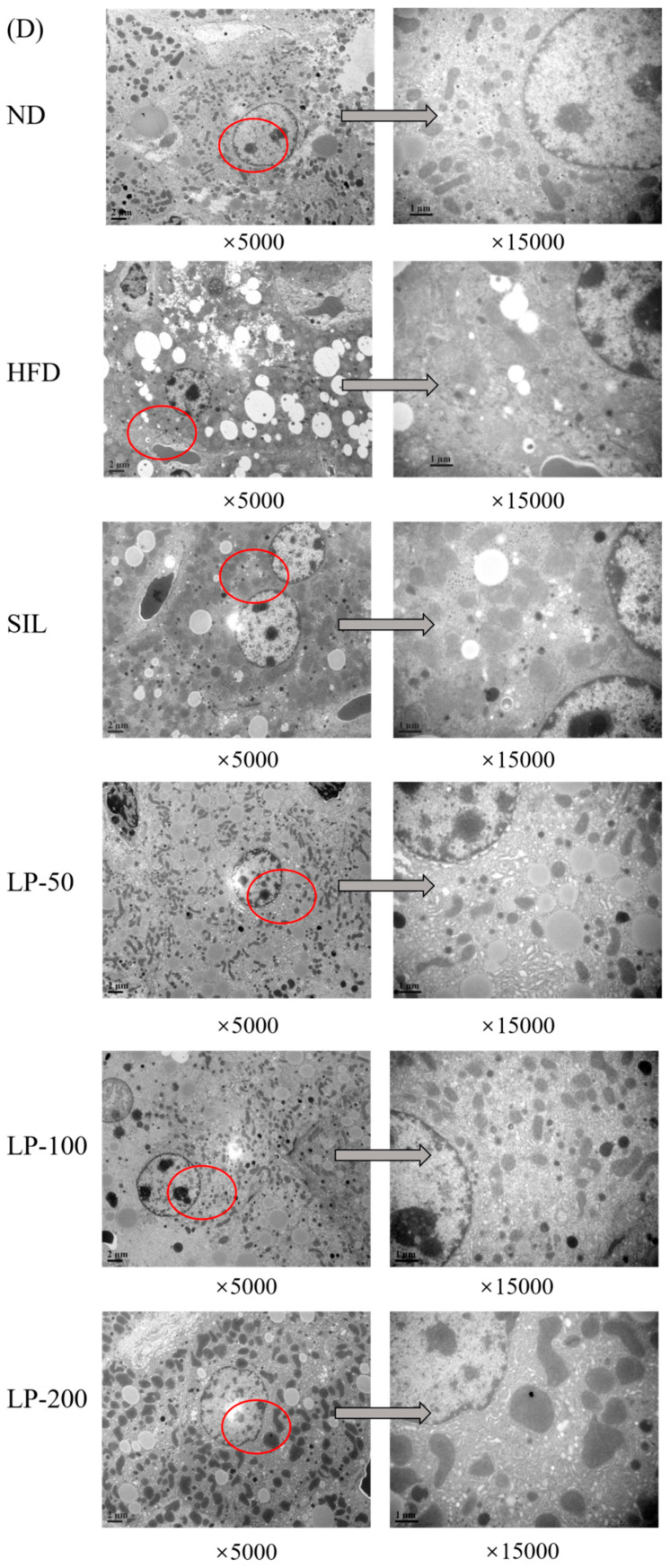

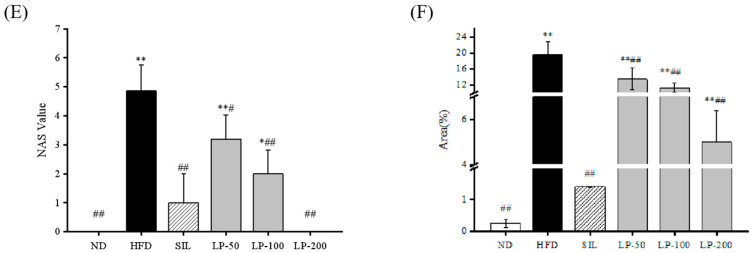
Micrograph (**A**), hematoxylin and eosin (H&E) staining (×400, scale bars of images are 20 μm) (**B**), oil red O staining (×200, scale bars of images are 50 μm) (**C**), transmission electron microscopy (×5000, scale bars of images are 2 μm; ×15,000, scale bars of images are 1 μm) (**D**) Histopathological analysis was performed using the NAFLD activity score (NAS) value for each group (**E**), and oil droplets were analyzed by Image J (**F**). Data are presented as means ± SD. Values with different labels are significantly different among the groups (* *p* < 0.05, ** *p* < 0.01 vs. ND group, ^#^ *p* < 0.05, ^##^ *p* < 0.01 vs. HFD group).

The results of the histopathological examination of the liver tissue are shown in Figure 5. The liver volume of the HFD group was enlarged, and white particles could be seen on the surface. In the SIL group and LPs groups, the size of the liver shrank, and the white particles of fat decreased (Figure 5A). Hematoxylin and eosin (H&E) staining revealed hypertrophy of the liver cells in the HFD group. The fat tissue was mainly composed of lipid droplets, and the hepatocyte cords were disordered. Compared with the HFD group, the LP-50 and LP-100 groups had fewer lipid droplets. The SIL and LP-200 groups greatly inhibited the accumulation of lipid droplets, attenuating the swelling and disorder of hepatocytes (Figure 5B). The oil red O staining and transmission electron microscopy (TEM) results were consistent with those of the H&E staining. In the ND group, there were no obvious lipid droplets in the hepatocytes. In contrast, there were many red lipid droplets in the model group. The numbers of these droplets in the LPs groups decreased, especially in the LP-200 group (Figure 5C). Hepatic steatosis is related to changes in the mitochondria of hepatocytes [31]. The results show that the electron density of lipid droplets was low, meaning the content of saturated fatty acids was high, and that the structure of hepatocytes in the model group was fuzzy with a blurry boundary. In the normal group, the amount of endoplasmic reticulum was reduced, and there were a large number of round lipid droplets of different sizes. After the intervention of SIL and LPs, the swelling degree of liposomes was reduced, the changes in the mitochondria and endoplasmic reticulum were significantly reduced, and the number of lipid droplets was significantly reduced. The electron density of liposomes was higher than that of the model group (Figure 5D). Livers also increased in size during HFD intervention and H&E staining showed macrovesicular steatosis, ballooning, and inflammation. According to the NAS scoring standard, the structural changes in the liver tissue in 10 visual fields were observed under a microscope, and the results in Figure 5E were obtained, which shows the NAS score: livers showed an average NAS score of 4.8. Quantitative analysis of oil red O staining showed that the fat area in the HFD group was 20%, while that in the LPs groups decreased to 14%, 11%, and 5%, respectively (Figure 5F).

### 2.8. Effects of LPs on the Protein Expressions of AMPK Pathways

**Figure 6:**
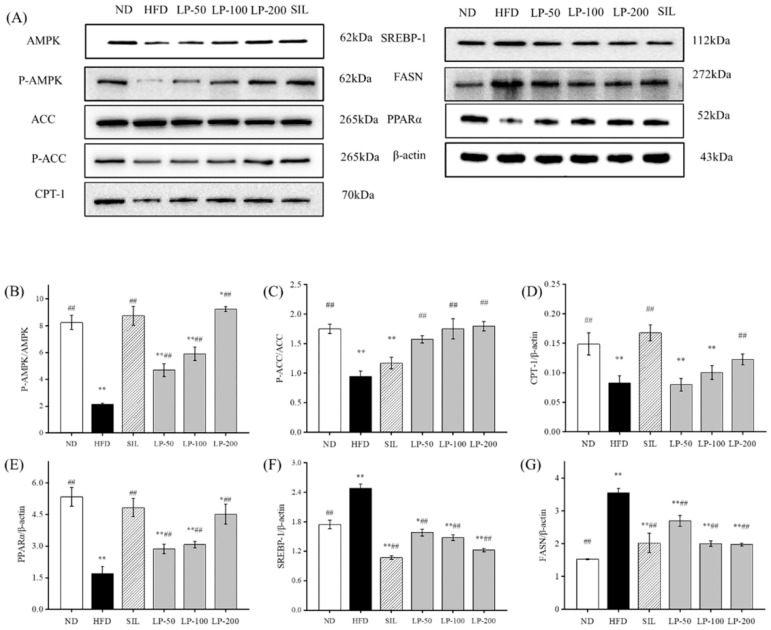
Effects of LPs on the AMP-activated protein kinase (AMPK) pathway-related proteins: (**A**) Western blot analysis of AMPK, phospho-AMP-activated protein kinase (p-AMPK), acetyl CoA carboxylase (ACC), p-ACC, carnitine palmitoyltransferase-1 (CPT-1), sterol regulatory element-binding transcription factor 1 (SREBP-1), fatty acid synthase (FASN), peroxisome proliferator-activated receptor alpha (PPARα), and β-actin. The expressions of p-AMPK (**B**), p-ACC (**C**), CPT-1 (**D**), PPARα (**E**), SREBP-1 (**F**), and FASN (**G**). Values are presented as means ± SD. Values with different labels are significantly different among the groups (* *p* < 0.05, ** *p* < 0.01 vs. ND group, ^##^ *p* < 0.01 vs. HFD group).

To clarify the molecular mechanism of LPs inhibiting the abnormal development of FFA, we examined the expression level of the AMPK pathway-related proteins. The phosphorylation level of the AMPK and ACC proteins in the HFD group was much lower than that in the ND group (*p* < 0.01, Figure 6). The expression of the sterol regulatory element-binding transcription factor 1 (SREBP-1) protein downstream was significantly up-regulated, resulting in a significant increase in fatty acid synthase (FASN) expression compared with the ND group (*p* < 0.01, Figure 6), as well as a significant decrease in the CPT-1 protein (*p* < 0.01). After the intervention of SIL and LPs, the phosphorylation levels of AMPK and ACC were significantly enhanced compared with the HFD group (*p* < 0.01, Figure 6), and the expression of the SREBP-1 protein down-stream was downregulated. Thus, the content of the FASN protein was decreased and the protein content of CPT-1 was increased. The expression of peroxisome proliferator-activated receptor alpha (PPARα) in the HFD group was significantly decreased (*p* < 0.01, Figure 6), and its expression was significantly upregulated by SIL and LPs (*p* < 0.01, Figure 6). These results show that LPs can inhibit the abnormal accumulation of FFA by regulating the synthesis of lipid metabolism-related proteins, thus increasing lipid β oxidation and reducing fatty acid synthesis.

### 2.9. Effects of LPs on the Expressions of Hepatic Nrf2 Pathway Proteins

**Figure 7:**
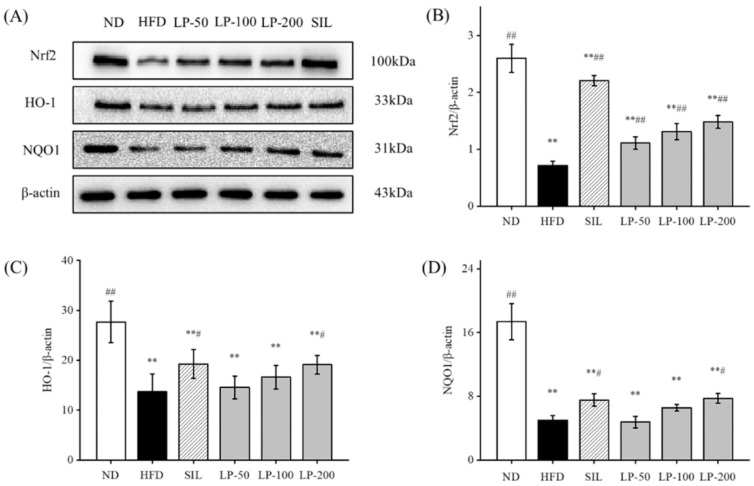
Effects of LPs on the Nrf2 pathway-related proteins: (**A**) Western blot analysis of Nrf2, HO-1, NQO1, and β-actin. The expressions of Nrf2 (**B**), HO-1 (**C**), and NQO1 (**D**). Values are presented as means ± SD. Values with different labels are significantly different among the groups (** *p* < 0.01 vs. ND group, ^#^ *p* < 0.05, ^##^ *p* < 0.01 vs. HFD group).

Studies have shown that upregulation of Nrf2 expression in the liver can improve NAFLD [32,33,34]. Therefore, we can speculate that LPs mitigate hepatic oxidative stress through the Nrf2 pathway. The expression levels of Nrf2, downstream HO-1, and NQO1 in the HFD group were significantly lower than the levels of those indicators in the ND group (*p* < 0.01, Figure 7). Compared with the HFD group, Nrf2 expression was significantly increased after SIL and LPs intervention (*p* < 0.01, Figure 7), and the expression levels of HO-1 and NQO1 in the SIL and LP-200 groups significantly increased (*p* < 0.05, Figure 7).

## 3. Discussion

In this study, we investigated the effects of LPs on liver metabolism. We found that LPs were able to lower body weight in LP-50, LP-100, and LP-200 groups by 24, 23, and 28 percent; to lower TG levels by 13, 29, and 45 percent; to lower LDL-c levels by 23, 41, and 51 percent; and to lower FFA levels by 28, 43, and 62 percent. After the administration of LPs, the markers for liver lesions were decreased, antioxidant capacities were enhanced, and inflammation inhibited. Upon further investigation, we found the mechanism of action to be via AMPK and Nrf2 pathways.

The conventional hypothesis regarding the pathogenesis of NAFLD is the “second hit” theory proposed by Day and James in 1998 [35]. In this theory, the “first strike” is liver fat deposition and hepatocyte fatty change in response to various lipid metabolism disorders. Insulin resistance (IR) is considered to be the central link of the first strike. The accumulation of fat in the liver is also related to a high-calorie diet, sedentary lifestyle, and possible genetic predisposition. The “second hit” follows, which mainly includes the effects of oxidative stress, lipid peroxidation, mitochondrial dysfunction, and increased production of serum endotoxin and inflammatory factors. This “second hit” increases the liver’s susceptibility to inflammatory necrosis and fibrosis, in turn accelerating the progression of NAFLD. Inflammation damage can also lead to non-alcoholic steatohepatitis, liver fibrosis, cirrhosis, and even liver cancer.

In recent years, marine active peptides have received growing attention due to their anti-hypertensive and anti-oxidative properties [36]. Mendisa et al. isolated bioactive peptides from squid and found them to inhibit lipid peroxidation by using a linoleic acid model [37]. In this study, functions of LPs were studied in a murine model of NAFLD, induced by an HFD. Excessive intake of fat leads to disorders of lipid metabolism in the liver, which can result in the clinical features of NAFLD such as fatty degeneration with increased serum FFA level and dyslipidemia [38,39]. As shown in Figure 1B and Figure 2, the liver index of the LPs and SIL groups decreased significantly compared with the HFD control group (*p* < 0.05). The TG, TC, LDL-C, and FFA levels significantly increased in the HFD group, and decreased in the experimental groups. AST and ALT are markers generally used for identifying liver lesions and dysfunctions and also can be used as indicators of NAFLD [40]. The levels of AST and ALT in the HFD group significantly increased (*p* < 0.01), indicating that liver damage had occurred in the HFD mice. The AST and ALT levels in the LPs group, however, significantly decreased (*p* < 0.05), suggesting that the effect of NAFLD on the liver was somewhat mitigated by the peptides.

Oxidative stress plays a critical role in the progression of NAFLD as the excess of reactive oxygen species directly induces lipid peroxidation [41]. MDA accumulation is used as a marker for oxidative stress as it is one of the major products of lipid peroxidation, and the markers of the endogenous antioxidant system that counteract this stress are T-AOC, GSH, SOD, and CAT [42]. Table 1 shows a significantly higher level of MDA in the HFD group compared to the ND group (*p* < 0.01), while T-AOC, GSH, SOD, and CAT levels were significantly decreased (*p* < 0.05). The level of MDA in the LPs group was improved. Based on these data, we determined that the protective effects of LPs in NAFLD are due to partially enhancing the endogenous antioxidant system and reducing the MDA level in hepatocytes, thus protecting the liver from excessive reactive oxygen species.

Inflammatory cytokines, also important regulators, are often overexpressed in NAFLD [43]. For example, IL-6 inhibits fat decomposition and promotes fat storage in NAFLD, while TNF-α inhibits the transport of lipids and lipoproteins, leading to an accumulation of lipids in liver cells [44]. In addition, TNF-α also mediates superoxide formation and lipid peroxidation, triggering a cascade of cytokine responses [45]. By examining the levels of TNF-α, IL-1β, and IL-6 in mice during our study, we found LPs to have an inhibitory effect on the HFD-induced inflammation.

H&E staining is a more intuitive approach to analyzing the characteristics of NAFLD, and oil red O staining can be used to show the accumulation of fat in liver tissues. Additionally, in this study, the alterations the in morphology of the liver cell mitochondria were observed by transmission electron microscopy. Figure 5 shows liver tissue from the HFD group in which yellow coloration, white fat granules, disordered arrangement of hepatocyte cords, fat vacuoles, and lipid accumulation can be seen. Compared with the HFD group, liver samples from the LPs group appeared ruddy and smooth, with neater hepatic cords, and a reduced amount of fat.

AMPK is a critical protein in energy regulation and *p*-AMPK reduces lipid synthesis through accelerating fatty acid β-oxidation. The AMPK-signaling pathway further affects liver lipid metabolism via downstream targets such as CPT-1 and FAS [46]. The FASN level is regulated by the inhibition of SREBP1 expression in response to AMPK phosphorylation, resulting in a decrease in lipid synthesis. AMPK also regulates lipid metabolism by adjusting the ACC and CPT-1 pathways, with previous studies having shown the role of PPAR-α in elevating fatty acid oxidation through the induction of CPT-1 expression [47]. The results of Figure 6 show that the HFD group had significantly reduced levels of p-AMPK, p-ACC, PPAR-α, and CPT-1, and significantly increased SREBP-1 and FASN content (*p* < 0.05). Notably, HFD generates mitochondrial dysfunction, and active PPAR-α negatively interferes with NF-κB activation [48]. Thus, liver PPAR-α downregulation in obesity also has a pro-inflammatory connotation. These outcomes indicate that in the HFD group, β-oxidation of fat in the liver was decreased and fatty acid synthesis was increased, leading to lipid accumulation in liver cells, eventually causing NAFLD. It can be seen that LPs altered the homeostasis of hepatic lipids and up-regulated the expression of the p-AMPK protein. A significant increase in PPAR-α and CPT-1 expression, and a significant decrease in the SREBP-1 and FASN content were seen in the LPs groups in a dose-dependent manner (*p* < 0.05). Thus, it can be said that LPs can effectively improve lipid metabolism in mice affected by NAFLD by decreasing fat synthesis, increasing fat oxidation, and inhibiting fat production. Recent studies demonstrated that AMPK regulated Nrf2 nuclear translocation to induce HO-1 gene expression [49]. Nrf2 plays a pivotal role in cellular defenses against oxidative stress by regulating the gene expression of antioxidant and detoxication enzymes [50].

Next, we measured the expression of Nrf2 and its associated pathways. As shown in Figure 7, regardless of the dosage, LPs are able to significantly increase the level of Nrf2. As well as this, the levels of HO-1 and NQO1 in mouse liver are increased. This indicates that AMPK affects the expression of HO-1 by regulating Nrf2. Therefore, LPs may ameliorate high-fat diet-related oxidative damage by activating the AMPK pathway to modulate the Nrf2-mediated antioxidant pathway.

## 4. Materials and Methods

### 4.1. Preparation of Monkfish Muscle Peptides

Monkfish (*Lophius litulon*) with an approximate body length of 39.21 ± 1.71 cm was purchased from a local market in Zhoushan, Zhejiang Province. Fish muscle peptides were obtained following an already described procedure [22]. Briefly, tissue fat was removed by adding 95% ethanol extraction followed by centrifugation. The precipitate was subsequently washed with distilled water and centrifuged again. The defatted monkfish meat was digested with neutral protease (E/S, 2000 U/g) at 45 °C for five hours. The hydrolysate was separated using a 1000 Da ultrafiltration membrane, and a peptide solution with a molecular weight of 1000 Da or less was collected. Free amino acids were removed by a 150 Da ultrafiltration membrane and were vacuum freeze-dried to obtain LPs with a molecular weight less than 1000 Da. Ultra-high pressure liquid chromatography and mass spectrometry (UPLC-MS) were performed. PEAKS studio was used to analyze the spectrum information, and the NCBI database was used for comparison and de novo sequencing analysis to obtain the structural composition of <1 kDa peptide.

### 4.2. Animals and Treatments

Six weeks old male ICR mice were provided by the Experimental Animal Center of Zhejiang Province. The Experimental Animal Ethics Committee of Zhejiang Ocean University approved the procedures for the use of the laboratory animals (Certificate Number SCXK ZHE2014-0001). Mice were housed in an animal facility at a room temperature of 20–25 °C and had free access to water and food. After seven days of adaptive feeding, the mice were randomly divided into the following six groups (48 in total, *n* = 8 per group): ND, HFD, HFD with silibinin (SIL, 80 mg/kg/day), HFD with LP-50 (50 mg/kg/day), HFD with LP-100 (100 mg/kg/day), and HFD with LP-200 (200 mg/kg/day). The HFD used was purchased from Research Diets, Inc. (New Brunswick, NJ, USA) (Product #D12492). NAFLD was induced by continuous feeding for eight weeks. Both LPs and silibinin were administered by intragastric needle feeding. After four weeks of intervention, the mice were fasted for sixteen hours before euthanization. Plasma was collected by centrifuging the blood at 4 °C and 4000 r/min for 10 min. The livers were acutely dissected and weighed.

### 4.3. Determination of the Serum Lipid Index, AST, ALT, and Liver Oxidative Stress Index

Serum was collected and analyzed for TC, FFA, LDL, TG, HDL, AST, ALT, MDA, CAT, T-AOC, GSH-Px, and SOD with the aid of detection kits according to the manufacturer’s instructions (Nanjing Jiancheng Biotechnology Research Institute, Nanjing, China).

### 4.4. Pro-Inflammatory Factors Analysis

The levels of hepatic TNF-α, IL-6, IL-Iβ, and IFN-γ were determined by Quantikine ELISA kits (Elabscience Biotechnology, Inc., Wuhan, China). The 96-well plates were coated with primary antibodies before the reference standards and test samples were added. After rinsing the plates, a biotinylated antibody was applied. A peroxidase-conjugated streptavidin was then added, and the concentrations of the primary antibodies were detected using Tetramethylbenzidine through analyzing OD at λ max = 450 nm.

### 4.5. Histopathological Examination of the Liver

Liver tissues were harvested and fixed overnight in 4% paraformaldehyde and then embedded in paraffin. Four-millimeter paraffin sections were subsequently made and stained with H&E. Photomicrographs were taken under an optical microscope (Biological microscope CX31, Olympus, Tokyo, Japan). TEM was performed to examine the detailed information of the liver injuries. Oil Red O staining was carried out according to the method used by Shen et al. [51]. Fresh liver sections were then taken and stained with an analytical kit (Nanjing Jiancheng Biotech, Nanjing, China). TEM procedure was determined according to Huang et al. [52].

### 4.6. Western Blotting

The liver tissue was homogenized using ultrasound and then lysed in a RIPA buffer. The procedure of Western blotting as described by Tang et al. was used to differentiate the protein molecules [53]. The protein concentration was measured using the bicinchoninic acid (BCA) method (KeyGENbio, Nanjing, China). Protein extracts were loaded onto 12% SDS-polyacrylamide gel and subsequently transferred onto a PVDF membrane. After incubating with primary and secondary antibodies, protein bands were visualized with enhanced chemiluminescence (ECL). Western blotting bands were imaged and quantitated with the FluorChem FC3 software (3.4.0.728, ProteinSimple, Waltham, MA, USA). All antibodies were purchased from Affinity (Affinity Biosciences, Cincinnati, OH, USA).

### 4.7. Statistical Analysis

Data were presented as mean ± standard deviation (SD) (*n* = 8). For comparing data in different groups, an ANOVA analysis was performed by the SPSS 19.0 software (IBM, Armonk, NY, USA). A probability value of less than 5% was considered statistically significant.

## 5. Conclusions

**Figure 8:**
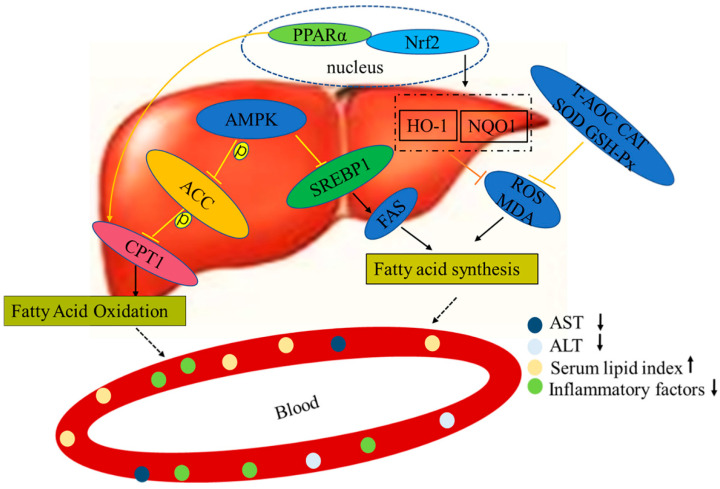
Mechanisms of LPs on alleviating NAFLD.

According to the results of our experiment, we hypothesize that LPs act by resisting the second attack of NAFLD pathogenesis (including oxidative stress, lipid peroxidation, and increased production of inflammatory factors), so as to assist in repairing liver function (Figure 8). We previously found that LPs had a high antioxidant activity and could effectively scavenge DPPH, hydroxyl, ABTS, and superoxide anion radicals, and protect RAW264.7 cells from H_2_O_2_-induced injury. LPs can be used as raw materials for natural antioxidants [26]. In order to provide a theoretical basis for the stable development and application of LPs and related products, we systematically analyzed the composition of LPs and identified the corresponding proteins using both database search and de novo sequencing methods. In the field of drug discovery, these functional proteins can be used as the lead compound for further modification and screening of active peptide analogues.

## Figures and Tables

**Table 1:** Effects of LPs on Antioxidant Capacities in Liver.

Groups	T-AOC (U/mg prot)	CAT (U/mg prot)	SOD (U/mg prot)	MDA (nmol/mg prot)	GSH-Px (U/mg prot)
ND	5.22 ± 0.07 ^##^	126.11 ± 1.37 ^##^	121.42 ± 0.97 ^##^	5.02 ± 0.28 ^##^	140.12 ± 1.63 ^##^
HFD	2.14 ± 0.47 **	50.26 ± 1.33 **	58.13 ± 1.01 **	9.03 ± 0.99 **	67.78 ± 1.67 **
SIL	5.10 ± 0.13 ^##^	123.03 ± 1.04 ^##^	111.79 ± 1.05 **^,^^##^	5.44 ± 0.14 ^#^	163.83 ± 2.39 **^,^^##^
LP-50	3.75 ± 0.13 **^,^^#^	73.22 ± 1.13 **^,^^##^	76.27 ± 0.88 **^,^^##^	7.66 ± 0.68 *	88.64 ± 2.27 **^,^^##^
LP-100	4.38 ± 0.17 **^,^^##^	95.55 ± 1.76 **^,^^##^	97.24 ± 0.84 **^,^^##^	6.16 ± 0.18 *^,^^#^	109.07 ± 1.12 **^,^^##^
LP-200	5.07 ± 0.15 ^##^	126.04 ± 1.97 ^##^	113.10 ± 0.47 **^,^^##^	4.92 ± 0.69 ^#^	135.41 ± 2.04 *^,^^##^

The data are expressed as the mean ± SD (*n* = 8 per group). ** *p* < 0.01 vs. ND group. ^##^ *p* < 0.01 vs. HFD group. * *p* < 0.05 vs. ND group. ^#^ *p* < 0.05 vs. HFD group. Groups: ND = normal control; HFD = HFD control; SIL = HFD + silibinin; LP-50 = HFD + 50 LP mg/kg; LP-100 = HFD + 100 LP mg/kg; LP-200 = HFD + 200 LP mg/kg.

## Data Availability

Not applicable.

## Supplementary Materials

The following supporting information can be downloaded at: https://www.mdpi.com/article/10.3390/md20050312/s1, Table S1: The free peptide was obtained by database search, Table S2: Free peptides with ALC% over 80 in de novo sequencing, Table S3: Source proteins of the identified peptides in Low Molecular Weight Monkfish (*Lophius litulon*) Peptides.
