# Supplementary material for: Monkfish Peptides Mitigate High Fat Diet-Induced Hepatic Steatosis in Mice
Source: Mar Drugs. 2022 May 5;20(5):312. doi: 10.3390/md20050312 (PMC9147042; doi:10.3390/md20050312)
Supplement: Supplementary file 1 [file marinedrugs-20-00312-s001.zip › marinedrugs-1697246-supplementary.pdf]

Supplementary

Table S1. The free peptide was obtained by database search

Protein Accession	Peptide	-10lgP	Mass	Length	m/z	z
332500944	L.ITGESGAG K.T	54.5	818.4134	9	410.2131	2
332500944	K.QKQREEQ AEPD.G	42.86	1356.627	11	679.3206	2
332500944	K.QREEQAE PD.G	41	1100.473	9	551.244	2
332500944	L.RNDNSSR F.G	36.86	994.458	8	498.2355	2
332500944	LTDRENQS. V	32.48	961.4465	8	481.7297	2
332500944	Q.KQREEQA EPD.G	32.25	1228.568	10	615.291	2
332500944	S.IAAVGGG GK.K	24.67	728.418	9	365.2175	2
332500944	R.NDNSSRF. G	22.02	838.3569	7	420.185	2
332500944	Q.REEQAEP D.G	21.05	972.4148	8	487.2145	2
156103922	L.ITGESGAG K.T	54.5	818.4134	9	410.2131	2
156103922	K.QKQREEQ AEPD.G	42.86	1356.627	11	679.3206	2
156103922	K.QREEQAE PD.G	41	1100.473	9	551.244	2
156103922	L.RNDNSSR F.G	36.86	994.458	8	498.2355	2
156103922	M.LTDRENQ S.V	32.48	961.4465	8	481.7297	2
156103922	Q.KQREEQA EPD.G	32.25	1228.568	10	615.291	2
156103922	S.IAAVGGG GK.K	24.67	728.418	9	365.2175	2
156103922	R.NDNSSRF. G	22.02	838.3569	7	420.185	2
156103922	Q.REEQAEP D.G	21.05	972.4148	8	487.2145	2
510950687	L.ITGESGAG K.T	54.5	818.4134	9	410.2131	2
510950687	K.QKQREEQ AEPD.G	42.86	1356.627	11	679.3206	2
510950687	K.QREEQAE PD.G	41	1100.473	9	551.244	2
510950687	L.RNDNSSR F.G	36.86	994.458	8	498.2355	2
510950687	M.LTDRENQ S.V	32.48	961.4465	8	481.7297	2

510950687	Q.KQREEQAEPD.G	32.25	1228.568	10	615.291	2
510950687	D.MLLVTNNP.Y	24.72	900.4739	8	451.2477	2
510950687	R.NDNSSRF.G	22.02	838.3569	7	420.185	2
510950687	Q.REEQAEPD.G	21.05	972.4148	8	487.2145	2
156103926	L.ITGESGAGK.T	54.5	818.4134	9	410.2131	2
156103926	K.QKQREEQAEPD.G	42.86	1356.627	11	679.3206	2
156103926	K.QREEQAEPD.G	41	1100.473	9	551.244	2
156103926	V.RNDNSSRF.G	36.86	994.458	8	498.2355	2
156103926	M.LTDRENQS.V	32.48	961.4465	8	481.7297	2
156103926	Q.KQREEQAEPD.G	32.25	1228.568	10	615.291	2
156103926	T.VRNDNSSRF.G	28.21	1093.527	9	547.77	2
156103926	R.NDNSSRF.G	22.02	838.3569	7	420.185	2
156103926	Q.REEQAEPD.G	21.05	972.4148	8	487.2145	2
156103924	L.ITGESGAGK.T	54.5	818.4134	9	410.2131	2
156103924	K.QKQREEQAEPD.G	42.86	1356.627	11	679.3206	2
156103924	K.QREEQAEPD.G	41	1100.473	9	551.244	2
156103924	V.RNDNSSRF.G	36.86	994.458	8	498.2355	2
156103924	M.LTDRENQS.V	32.48	961.4465	8	481.7297	2
156103924	Q.KQREEQAEPD.G	32.25	1228.568	10	615.291	2
156103924	T.VRNDNSSRF.G	28.21	1093.527	9	547.77	2
156103924	R.NDNSSRF.G	22.02	838.3569	7	420.185	2
156103924	Q.REEQAEPD.G	21.05	972.4148	8	487.2145	2
332500904	L.ITGESGAGK.T	54.5	818.4134	9	410.2131	2
332500904	K.QKQREEQAEPD.G	42.86	1356.627	11	679.3206	2
332500904	K.QREEQAEPD.G	41	1100.473	9	551.244	2
332500904	A.RNDNSSRF.G	36.86	994.458	8	498.2355	2

332500904	Q.KQREEQAEPD.G	32.25	1228.568	10	615.291	2
332500904	S.IAAVGGGGK.K	24.67	728.418	9	365.2175	2
332500904	R.NDNSSRF.G	22.02	838.3569	7	420.185	2
332500904	Q.REEQAEPD.G	21.05	972.4148	8	487.2145	2
332500946	L.ITGESGAGK.T	54.5	818.4134	9	410.2131	2
332500946	K.QKQREEQAEPD.G	42.86	1356.627	11	679.3206	2
332500946	K.QREEQAEPD.G	41	1100.473	9	551.244	2
332500946	V.RNDNSSRF.G	36.86	994.458	8	498.2355	2
332500946	Q.KQREEQAEPD.G	32.25	1228.568	10	615.291	2
332500946	T.VRNDNSSRF.G	28.21	1093.527	9	547.77	2
332500946	R.NDNSSRF.G	22.02	838.3569	7	420.185	2
332500946	Q.REEQAEPD.G	21.05	972.4148	8	487.2145	2
156103928	L.ITGESGAGK.T	54.5	818.4134	9	410.2131	2
156103928	K.QKQREEQAEPD.G	42.86	1356.627	11	679.3206	2
156103928	K.QREEQAEPD.G	41	1100.473	9	551.244	2
156103928	L.RNDNSSRF.G	36.86	994.458	8	498.2355	2
156103928	Q.KQREEQAEPD.G	32.25	1228.568	10	615.291	2
156103928	R.NDNSSRF.G	22.02	838.3569	7	420.185	2
156103928	Q.REEQAEPD.G	21.05	972.4148	8	487.2145	2
510950689	L.ITGESGAGK.T	54.5	818.4134	9	410.2131	2
510950689	K.QKQREEQAEPD.G	42.86	1356.627	11	679.3206	2
510950689	K.QREEQAEPD.G	41	1100.473	9	551.244	2
510950689	L.RNDNSSRF.G	36.86	994.458	8	498.2355	2
510950689	Q.KQREEQAEPD.G	32.25	1228.568	10	615.291	2
510950689	R.NDNSSRF.G	22.02	838.3569	7	420.185	2
510950689	Q.REEQAEPD.G	21.05	972.4148	8	487.2145	2

1786483305	M.IGAPDMA FPR.M	35.38	1073.533	10	537.7726	2
1786483305	A.GASVDLT. I	20.92	661.3282	7	662.3401	1
288909106	F.LVSDVAIL .T	28.41	828.4957	8	415.2537	2
288909106	E.NKALGLT	23.84	715.4229	7	358.7202	2
288909106	P.FIIAALT.I	22.47	747.4531	7	374.7337	2
33313529	K.IIAPPERK Y.S	23.15	1085.623	9	543.8176	2
288908941	Y.TKTLTISP N(+.98)N.L	21.23	1088.571	10	545.288	2
288909146	M.MVSIGLN Q.P	28.17	860.4426	8	431.2262	2
289183130	A.EGVETVT. T	21.05	733.3494	7	367.684	2
288909148	K.DLLGFMA VLL.A	23.19	1090.61	10	546.313	2
289183312	I.KTYLVLFL LT.L	22.42	1209.737	10	605.8715	2
288908968	Q.EAIWLVL. P	23.31	842.4902	7	422.2497	2
685824075	D.LVGFAAIL .I	20.84	802.4952	8	402.2549	2
288908809	G.LAGATAL. M	20.33	615.3591	7	616.3657	1
288908765	A.LVAGGVV FSVML.L	23.68	1190.673	12	596.3397	2
288909094	A.LAGGPPG V	20.3	666.3701	8	334.1942	2
289183172	K.LAALSVTI .L	22.88	786.4851	8	394.2495	2
288909061	L.MTQRGPA P.L	21.7	856.4225	8	429.2217	2
1786483282	L.QSSTGTL. S	28.25	692.3341	7	347.1762	2
288908699	T.MPVVAIL T.A	32.35	842.4935	8	422.2517	2
25005627	A.Q(+.98)VLI PLMPVTAF.I	21.99	1328.741	12	665.3802	2
288909016	H.VIIGSIF.L	21.47	747.4531	7	748.4601	1
745791721	R.LATIPAGF S.K	22.96	875.4752	9	438.7425	2

Table S2. Free peptides with ALC% over 80 in de novo sequencing

Peptide	ALC (%)	length	m/z	z	Mass
PPLLE	97	5	568.3333	1	567.3268
FLRLL	94	5	331.2231	2	660.4323
LLVVV	93	5	542.3905	1	541.3839
FCPPP	93	5	560.2563	1	559.2464
WRPPL	92	5	334.6971	2	667.3806
KAAGF	90	5	493.276	1	492.2696

LVRLL	90	5	307.2228	2	612.4323
MLLLR	90	5	323.2083	2	644.4044
TERGY	90	5	313.1501	2	624.2867
LLVY	90	4	507.3167	1	506.3104
LLRLL	89	5	314.2311	2	626.4479
PPLLE	88	5	568.3333	1	567.3268
LLVGRPR	88	7	405.7683	2	809.5235
SSLAAKK	88	7	352.7215	2	703.4228
VVRLLP	87	6	348.7411	2	695.4694
AAFVVP	87	6	302.1761	2	602.3428
PPLLPR	87	6	346.726	2	691.438
LLRLF	86	5	331.2231	2	660.4323
LLRLL	86	5	314.2308	2	626.4479
RLLLR	85	5	335.7397	2	669.465
WGAAGGGVP	85	9	386.1911	2	770.3711
LLAW	85	4	502.3018	1	501.2951
VVLL	84	4	443.322	1	442.3155
RVLPR	84	5	320.7157	2	639.418
LLVVV	84	5	542.3905	1	541.3839
LLRLL	84	5	314.2307	2	626.4479
MLRLP	83	5	315.193	2	628.373
VLLK	83	4	472.3486	1	471.342
FLTL	83	4	493.3013	1	492.2948
KKLLF	83	5	648.4438	1	647.437
LLPLPP	83	6	325.2177	2	648.421
FLFLR	82	5	348.2146	2	694.4166
RLLLR	82	5	335.7392	2	669.465
LLVVR	82	5	300.2134	2	598.4166
LLAL	82	4	429.3066	1	428.2999
LDLLLR	82	6	371.7443	2	741.4749
RAAHGKAPR	81	9	482.2811	2	962.5522
LFLLR	81	5	331.2223	2	660.4323
LLVVLK	81	6	342.7538	2	683.4945
KGSS	81	4	378.2019	1	377.191
KLPL	81	4	470.3328	1	469.3264
KKST	81	4	463.2908	1	462.2802
KLLLLK	81	6	364.275	2	726.5367
KKLHLT	81	6	370.2443	2	738.4752
RSPGPP	81	6	610.3248	1	609.3234
FLLLR	81	5	331.2231	2	660.4323
LDLLR	81	5	315.2018	2	628.3908
WLLRL	81	5	350.7278	2	699.4432
VVVS	81	4	403.255	1	402.2478
LLVLR	81	5	307.223	2	612.4323
FGGPK	80	5	505.2766	1	504.2696
KKLFGGKKK	80	9	517.347	2	1032.681
KAAR	80	4	445.2907	1	444.2808
RVLLPR	80	6	377.2574	2	752.5021
QPAAP	80	5	483.2544	1	482.2489
KAAR	80	4	445.2907	1	444.2808
FLRVL	80	5	324.2151	2	646.4166
RALLL	80	5	585.4072	1	584.4009
VLKL	80	4	472.3485	1	471.342

PGPPRGGKP	80	9	431.7477	2	861.4821
TLRLF	80	5	325.2043	2	648.3959
KKLVNPL	80	7	406.2751	2	810.5327
PLRLLP	80	6	354.7415	2	707.4694
HLPGSP	80	6	607.3192	1	606.3126
MPAAKP	80	6	614.3292	1	613.3257
EAPKAPP	80	7	709.3912	1	708.3806

Table S3. Source proteins of the identified peptides in Low Molecular Weight Monkfish (Lophius litulon) Peptides

Protein ID	-10lgP	Area Sample 1	Avg. Mass	Description
2	89.6	52500000	29575	cardiac muscle myosin heavy chain 6 alpha partial [Lophius vaillanti]
3	89.6	52500000	30142	cardiac muscle myosin heavy chain 6 alpha partial [Lophius americanus]
5	89.6	58303000	30920	cardiac muscle myosin heavy chain 6 alpha partial [Antennarius striatus]
6	89.6	50648000	30269	cardiac muscle myosin heavy chain 6 alpha partial [Himantolophus albinares]
7	89.6	50648000	30170	cardiac muscle myosin heavy chain 6 alpha partial [Oneirodes macrosteus]
8	89.6	40159000	28355	cardiac muscle myosin heavy chain 6 alpha partial [Chaunax suttkusi]
9	89.6	38308000	27492	cardiac muscle myosin heavy chain 6 alpha partial [Melanocetus johnsonii]
10	89.6	36933000	28238	cardiac muscle myosin heavy chain 6 alpha partial [Ogcocephalus parvus]
11	89.6	36933000	29289	cardiac muscle myosin heavy chain 6 alpha partial [Ogcocephalus nasutus]
138	0	20071000	56940	cytochrome c oxidase subunit 1 (mitochondrion) [Oneirodes bradburyae]
23	0	30839000	42599	cytochrome b (mitochondrion) [Acentrophryne dolichonema]
1336	0	202760000	6313	beta-actin partial [Lophius piscatorius]
944	0	357540	37657	NADH dehydrogenase subunit 2 (mitochondrion) [Puck pinnata]
453	0	7732300	67297	NADH dehydrogenase subunit 5 (mitochondrion) [Malthopsis jordani]
522	0	2234500	67027	NADH dehydrogenase subunit 5 (mitochondrion) [Lophiodes caulinaris]
547	0	0	42160	cytochrome b (mitochondrion) [Malthopsis jordani]
550	0	1104600	66940	NADH dehydrogenase subunit 5 (mitochondrion) [Gigantactis vanhoeffeni]
615	0	3566800	35419	NADH dehydrogenase subunit 1 (mitochondrion) [Bertella idiomorpha]
632	0	20668000	41905	cytb partial (mitochondrion) [Lophiodes reticulatus]
655	0	1252600	50417	NADH dehydrogenase subunit 4 (mitochondrion) [Coelophrys brevicaudata]
706	0	0	37798	NADH dehydrogenase subunit 2 (mitochondrion) [Tetrabrachium ocellatum]

719	0	1595200	35454	NADH dehydrogenase subunit 1 (mitochondrion) [Acentrophryne dolichonema]
637	0	91358000	67522	NADH dehydrogenase subunit 5 (mitochondrion) [Thaumatichthys pagidostomus]
981	0	3945900	50833	NADH dehydrogenase subunit 4 (mitochondrion) [Gigantactis vanhoeffeni]
824	0	1648300	51022	NADH dehydrogenase subunit 4 (mitochondrion) [Ceratias sp. CU44E]
809	0	9650200	25156	ATPase subunit 6 (mitochondrion) [Lophiodes caulinaris]
1469	0	0	25173	ATPase subunit 6 (mitochondrion) [Lophiomus setigerus]
885	0	28596000	29531	cytochrome c oxidase subunit III (mitochondrion) [Centrophryne spinulosa]
989	0	229680	39050	short-wavelength sensitive opsin 2A alpha [Lophius vaillanti]
